# On the repulsive interaction between localised vegetation patches in scarce environments

**DOI:** 10.1038/s41598-020-62677-6

**Published:** 2020-04-01

**Authors:** E. Berríos-Caro, M. G. Clerc, D. Escaff, C. Sandivari, M. Tlidi

**Affiliations:** 1 Departamento de Física and Millennium Institute for Research in Optics, Facultad de Ciencias Físicas y Matemáticas, Universidad de Chile, Casilla, 487-3 Santiago Chile; 2 Complex Systems Group, Facultad de Ingeniería y Ciencias Aplicadas, Universidad de los Andes, Avenida Monseñor álvaro del Portillo No 12.455, Las Condes, Santiago Chile; 3 Département de Physique, Faculté des Sciences, Université Libre de Bruxelles (U.L.B.), CP 231, Campus Plaine, B-1050 Bruxelles, Belgium

**Keywords:** Ecological modelling, Nonlinear phenomena

## Abstract

Fragmentation followed by desertification in water-limited resources and/or nutrient-poor ecosystems is a major risk to the biological productivity of vegetation. By using the vegetation interaction-redistribution model, we analyse the interaction between localised vegetation patches. Here we show analytically and numerically that the interaction between two or more patches is always repulsive. As a consequence, only a single localised vegetation patch is stable, and other localised bounded states or clusters of them are unstable. Following this, we discuss the impact of the repulsive nature of the interaction on the formation and the selection of vegetation patterns in fragmented ecosystems.

## Introduction

The formation of localised structures is a ubiquitous pattern forming phenomenon in nature. It has been experimentally observed and theoretically described in almost all fields of natural science, such as biology, nonlinear photonics, physics, plant ecology, and medicine (see overviews^1–9,10^). They are aperiodic and spatially localised patterns consisting of isolated spots surrounded by regions in the uniform state. Two types of localised structures can be distinguished depending whether their exponentially decaying tails possess or are devoid of spatial oscillations. In the former case, the interaction between spots alternates between attraction and repulsion depending on the distance separating the spots. This allows for the stabilisation of bounded states and clusters of spots^11–14^. However, when the tail decreases monotonically, i.e., without intrinsic oscillations, the interaction between localised spots can be either purely attractive^15^ or repulsive^16^. In this case, bounded localised structures are excluded.

In the field of plant ecology, localised structures consist either of localised vegetation patches, randomly distributed on bare soil^17–20^, or on the contrary consist of localised spots of bare soil randomly distributed on a uniform cover^21^. Their formation is attributed to the fact that the facilitative interaction occurs over a small spatial scale compared to the competitive one^22,23^. The competition tends to inhibit the growth of vegetation on a long spatial range namely because of water and/or nutrient scarcity while the facilitation interaction acts on a short spatial range.

## Results

The interaction between localised vegetation patches in arid and semi-arid landscapes has received only limited attention^21^. We show that the interaction between localised vegetation patches is always repulsive. Indeed, bounded states of vegetation patches are not allowed. We demonstrate that the repulsive nature of this interaction and the boundary conditions allow for the coexistence of several vegetation patterns with different wavelengths. In particular, the repulsive interaction strongly affects the pattern formation and the pattern selection processes by stabilising hexagon, square, and superlattices type of patterns. Likewise, the dynamical law governing the repulsive interaction between spots is established analytically and confirmed by numerical simulations.

**Figure 1:**
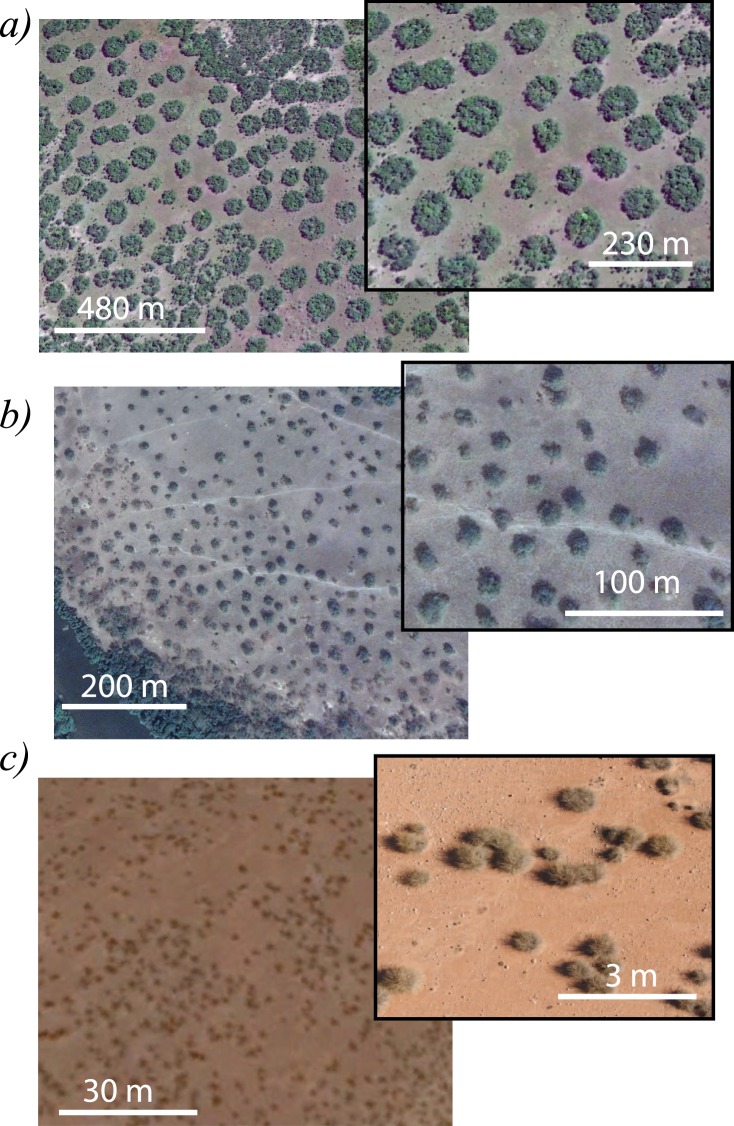
Satellite snapshots of self-organisation of localised patches obtained using Google Earth Pro. (**a**) Zambia, Southern Africa ($1{3}^{\circ }46{\prime} 49.07{\prime\prime}$S, $2{5}^{\circ }16{\prime} 56.97{\prime\prime}$E). (**b**) Ivory Coast, West Africa (${7}^{\circ }14{\prime} 53.01{\prime\prime}$N, ${6}^{\circ }06{\prime} 27.83{\prime\prime}$W). (**c**) Morocco, North West Africa ($3{3}^{\circ }06{\prime} 24.02{\prime\prime}$N, ${4}^{\circ }22{\prime} 01.90{\prime\prime}$W). The insets account for enlarged snapshots of the respective zones.

Patchy landscapes driven by either water scarcity and/or nutrient-poor territories are a rule rather than the exception in arid and semi-arid ecosystems. Examples of patchy landscapes of the African, American, Asian, and Australian continents are shown in Fig. 1. They are generically called vegetation patterns^22,23^. Either water limited resources and/or nutrient-poor territories characterise these landscapes. In the former case, the potential evaporation and transpiration of the plants exceed the water supply provided by rainfalls. At the level of an individual plant, the water scarcity provokes hydric stress that affects both the plant survival capacity and the plant growth. At the community level, this hydric stress promotes clustering behaviour, which induces spatial landscape fragmentation. It is now generally understood that this adaptation to hydric stress involves a symmetry-breaking instability leading to the establishment of a stable spatial pattern^24–26^. Arid vegetation patterning phenomenon is not specific to particular plants or soils. Vegetation may wholly consist of grasses, shrubs, and trees. They may develop on ground ranging from sandy and silty to clayey. This can be observed in different spatial scales, which vary from hundreds to tens of centimetres (cf. Fig. 1). Vegetation patterns arise via a Turing-like instability^27^ that acts even under strictly homogeneous and isotropic environmental conditions. Vegetation patterns are stabilised, thanks to the balance between positive and negative interactions^28–31^. The pattern wavelength then results from an interplay between short-range facilitation and long-range competition^22,23^. Other modelling approaches based on reaction-diffusion models that incorporate water dynamics have been proposed to explain arid vegetation pattern formation that are based on this spatial symmetry breaking mechanism^32–34^. The role of climatic fluctuations as a source of noise-induced symmetry breaking instability has also been reported^35,36^. Earlier reports using cellular automata to investigate the influence of water redistribution and plant growth on vegetation pattern formation have been proposed^37–41^.

### Vegetation model

For a strictly isotropic and homogeneous environment, the biomass *b*(**r**, *t*) evolves according to the logistic equation in which we incorporate the facilitative and competitive nonlocal interaction between individual plants^21–23^. This modelling approach explains several dynamical behavior such as bands often called vegetation stripes^22,23^, localised vegetation patches^42,43^, gaps^24,25^, and fairy circles^14,44^. Our analysis is based on the interaction-redistribution model for vegetation in arid landscapes

1 $${\partial }_{t}b({\bf{r}},t)={M}_{f}({\bf{r}},t)(1-b)b-\mu {M}_{c}({\bf{r}},t)b+D{\nabla }^{2}b,$$

where

2 $${M}_{f,c}({\boldsymbol{r}},t)\equiv exp\left({\chi }_{f,c}\int {\Phi }_{f,c}({\boldsymbol{r}}{\prime} )b({\boldsymbol{r}}+{\boldsymbol{r}}{\prime} ,t)d{\boldsymbol{r}}{\prime} \right),$$

with ***r*** and *t* being the spatial coordinates and time, respectively. The nonlocal factors *M*_*f*_ and *M*_*c*_ account for facilitation and competition mechanisms of the plant-to-plant feedbacks, respectively. The plant-to-plant feedbacks are given by the influence functions Φ_*f*,*c*_(**r**). The facilitation kernel Φ_*f*_ depends on the overground canopy which can provide shelter for other plants to grow. Conversely, the competition kernel Φ_*c*_ depends on the root sphere size which depletes ground resources, preventing other vegetation to grow. The strength of the facilitation and competition processes are, respectively, characterised by *χ*_*f*_ and *χ*_*c*_ parameters. *μ* accounts for the aridity parameter, which is mainly related to rainfall supplied to the land surface. Finally, *D* is the rate at which biomass diffuse.

**Figure 2:**
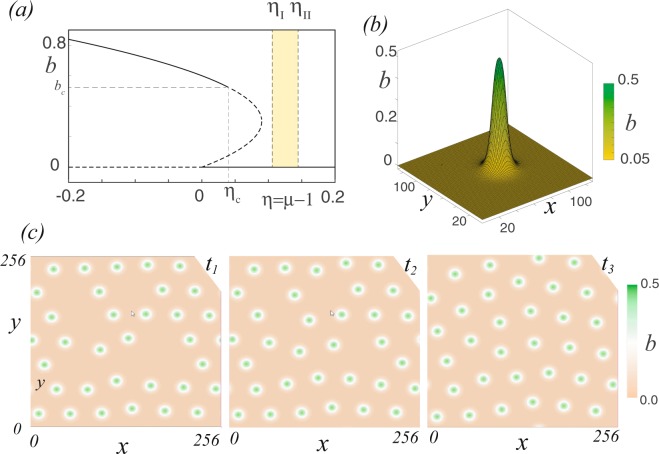
Localised vegetation patches in model Eq. (1). (**a**) Typical bifurcation diagram (*χ*_*f*_ > 1 + *χ*_*c*_), biomass *b* as function of effective aridity *η* = *μ* − 1. *η*_*c*_ stands for to the spatial instability threshold. *η*_*I*_ and *η*_*II*_ delimit the region where localised vegetation patches are observed. (**b**) Localised vegetation patch obtained from numerical simulation of model Eq. (1) for *μ* = 1.04, *χ*_*f*_ = 2, *χ*_*c*_ = 1, *D* = 1, ${\Phi }_{f,c}({\bf{r}})={e}^{-| {\bf{r}}| /{\sigma }_{f,c}}/{\sigma }_{f,c}$, *σ*_*c*_ = 4.5, and *σ*_*f*_ = 0.05. (**c**) Temporal evolution of localised vegetation patches (*t*_1_ < *t*_2_ < *t*_3_), from a random initial distribution of localised vegetation patches.

When the aridity is large enough, model Eq. (1) only exhibits as a uniform equilibrium the barren soil state (*b* = 0). In the case of long-range competition and short-range facilitation when the aridity is decreased, model Eq. (1) exhibits coexistence between barren and uniform vegetation cover state, which corresponds to a bistability region. By further decreasing the aridity, the barren soil state becomes unstable, and only the uniform vegetation cover state persists as a stable state. Figure 2 shows the typical bifurcation diagram of the model Eq. (1). Starting with the uniform vegetation cover state, with an increase in aridity, due to the plant-to-plant interaction (facilitation and competition), this solution presents a spatial spontaneous symmetry breaking giving rise to vegetation patterns at *b* = *b*_*c*_. These solutions persist beyond the tipping point. When hydric stress is further increased, patterns are replaced by isolated vegetation patches. The region where the localised patches are observed is represented by the painted region in Fig. 2a. The typically localised vegetation patch exhibited by model Eq. (1) is shown in Fig. 2b. A single patch surrounded by a bare state is stable in the parameter range *η*_*I*_ < *η* < *η*_*II*_. These localised solutions are characterised to have a bell shape without any damped oscillatory tails. In the region of localised patches, if one starts with a large number of patches initially randomly placed, one can observe how the interaction between localised vegetation patches leads to the self-organisation of these structures (see Fig. 2c), which tends to evolve into a pattern vegetation cover. To survive, plants have to self-organise in order to optimise the use of scarce resources.

To understand the dynamics of the vegetation patches and due to the complexity of the nonlocal vegetation model Eq. (1), we explore the neighbourhood of the transition point of the barren soil state, and assume that biomass varies slowly spatially. That is, for an idealised situation of a strictly isotropic and homogeneous environment from model Eq. (1) ^22,23^, the biomass density *b*(**r**, *t*) = *ρ*(**r**, *t*) ≪ 1 evolves according to the dimensionless kinetic equation, (*the interaction-redistribution model*)^21^

3 $${\partial }_{t}\rho =-\rho (\eta -\kappa \rho +{\rho }^{2})+(D-\Gamma \rho ){\nabla }^{2}\rho -\alpha \rho {\nabla }^{4}\rho ,$$

where *η* ≡ *μ* − 1 accounts for the aridity, *κ* ≡ *χ*_*f*_ − *χ*_*c*_ + 1 stands for the difference between the interaction strengths associated with the competitive and facilitative processes. This parameter is often called the cooperativity. The parameters Γ and *α* are the nonlinear diffusion coefficients that are determined by the influence functions (an explicit derivation is provided in^21^).

The homogenous steady state solutions of (3) are explicitly given by *ρ* = 0 and ${\rho }_{\pm }=(\kappa \pm \sqrt{{\kappa }^{2}-4\eta })/2$. The bare state is *ρ*_0_ = 0. The solutions *ρ*_±_ correspond to uniform plant distributions. When the strength of the competitive interaction is larger than the one associated with facilitative interaction, the cooperativity parameter *κ* becomes negative, the homogeneous cover biomass *ρ*_+_ decreases monotonously with the aridity parameter *η*, and the solution *ρ*_−_ does not physically exist. This is because the biomass should be real and positive. The state *ρ*_−_ only exists then if the cooperativity parameter *κ* is positive. It corresponds to the occurrence of a tipping point and, therefore, bistability between the homogeneous cover and bare state. In addition to the homogenous cover and the bare state solutions, the model Eq. (3) supports localised patches^17^, and gaps^21^. Recently, it has been shown that localised gaps exhibit a homoclinic snaking type of behaviour^45^.

**Figure 3:**
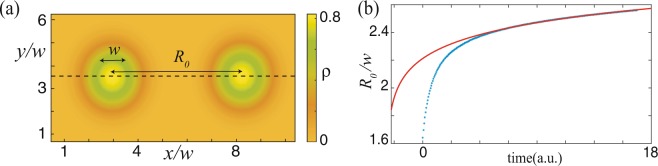
Interaction of two localised patches, top panels interaction-redistribution model Eq. (3). (**a**) Two localised patches separates by *R*_0_(*t*) distance. (**b**) Temporal evolution of the distance between the localised patches *R*_0_(*t*) with periodic boundary conditions. The dotted line corresponds to numerical data, while the solid line to the curve fitting by equation ${R}_{0}(t)/w=(1/\gamma )\,ln\,(At)$, with *γ* and *A* as fitting parameters. Simulation parameters used are *η* = 0.12, *κ* = 0.6, Δ = 0.02, Γ = 0.5, and *α* = 0.125.

For a moderate level of aridity, a single spot may exhibit a self-replication phenomenon that tends to repopulate the whole ecosystem^46^. However, for large values of the aridity level, a single spot is stable^17^. The single localised patch is a stable vegetation structure characterised by monotonously decreasing tails. The spatial damped oscillations around the bare state, i.e., *ρ* = 0, are physically forbidden since the biomass is a positively defined quantity. If two localised patches are close to one another, they interact through their exponentially decaying tails. In this case, bounds of localised patches and clusters of them are unstable on a long-time evolution. An example of such behaviour is illustrated in Fig. 3. From this figure, we can infer that the interaction between vegetation spots is repulsive. Bounded localised patches reported in^17^ are not possible for a long time since the interaction between patches is always repulsive.

### Interaction between localised vegetation patches

The interaction of two or more localised states through their overlapping decaying oscillatory tails has been investigated theoretically^11–15,21^. In this case, depending on the distance separating the two spots, the interaction can be attractive or repulsive. Likewise, there are cases where the interaction between localised states is purely attractive^15^. In our case, the interaction is always repulsive irrespective of the distance separating the spots. In what follows, we provide an analytical understanding of the interaction between two localised spots. To derive the interaction law, we use the interaction-redistribution model Eq. (3). For this purpose, we add a small perturbation to the linear superposition of the two separated spots as

4 $$\rho ({\bf{r}},t)={\rho }^{-}\left({\bf{r}}+\frac{{R}_{0}(t)}{2}\widehat{r}\right)+{\rho }^{+}\left({\bf{r}}-\frac{{R}_{0}(t)}{2}\widehat{r}\right)+P,$$

where ${\rho }^{\pm }\left(\overrightarrow{r}\mp {R}_{0}/2\ \widehat{r}\right)$ is a localised patch of vegetation located at  ± *R*_0_/2$\widehat{r}$, and ${\bf{r}}=r\widehat{r}$ accounts for the spatial coordinates, with $\widehat{r}$ and *R*_0_(*t*) the unitary vector and the distance between localised patches, respectively. We choose the coordinate system in such a way that its origin is located at the mid-point between the two localised patches. $P=P(\overrightarrow{r},{R}_{0}(t))$ is a small correction function. Introducing the above ansatz in Eq. (3), linearizing in *P*, applying the solvability condition, and after straightforward calculations, we derive the following spots interaction law (see methods section and ref. ^47^)

5 $${\dot{R}}_{0}=C\frac{{e}^{-\sqrt{\eta /D}{R}_{0}}}{\sqrt{{R}_{0}}},$$

where *C* is a positive constant determined by numerical integration. Hence, the interaction is repulsive by nature. The kinetic Eq. (5) describes long-range localised vegetation patches interaction. From this equation, we see that the interaction decays monotonously as a function of the distance separating two spots. Note that the level of aridity *η* and the diffusion coefficient *D* influence the interaction law Eq. (5). Indeed, more aridity and less diffusion tends to decrease the strength of the interaction. Figure 3b shows quite a fair agreement between the numerical simulation of model Eq. (3) and analytical results obtained by formula (5). From an ecological point of view, in the spatial region in between the two localised patches, the competition for resources (water or nutrients) through their lateral root spreads is strong. This theoretical prediction explains why two localised patches should repel in order to optimise water or nutrient uptake.

### Stabilization of squares and superlattices

**Figure 4:**
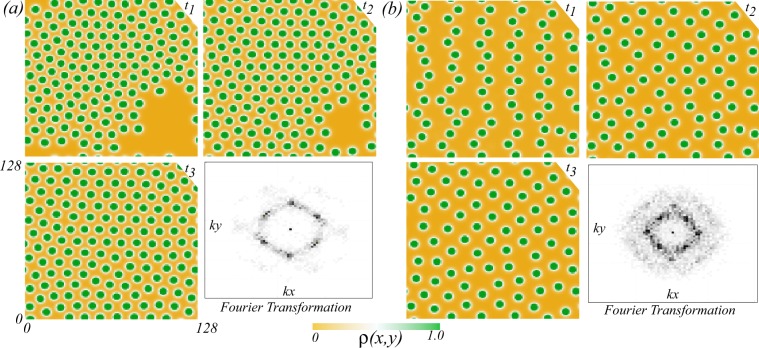
Temporal evolution of localised patches lattice of the interaction-redistribution model Eq. (3) with periodical boundary conditions, different initial distributions of localised spots (*t*_1_ < *t*_2_ < *t*_3_), and respective Fourier transform of the last state. Parameters are *η* = 0.5, *κ* = 1.0, *D* = 0.6, Γ = 3, and *α* = 1. The density of localised vegetation patches high (**a**) and low (**b**). For the high and the low density of localised spots, the tendency is to a hexagonal or square lattice, respectively.

In most of the spatially extended systems, close to the Turing instability threshold, the generic sequence Hexagons *π*-stripes (labyrinth)-Hexagons 0 has been established in^48^. The relative stability analysis has shown that the squares are always unstable^49^. Far from the Turing instability, numerical simulations of the interaction-redistribution model Eq. (3) with periodic boundary conditions show that the hexagonal structures are formed with a high density of localised spots, as shown in Fig. 4(a). This structure is obtained by using an initial condition consisting of a cluster of localised patches (*t*_1_). Due to the repulsion between the vegetation spots, these localised states invade the available space (*t*_2_), tending finally to form a hexagonal lattice (*t*_3_). The above is reaffirmed, by calculating the Fourier transform of the last state. Every single spot can be seen as a particle interacting with its neighbourhood spot in a repulsive way. The pattern depicted in Fig. 4a is expected since it corresponds to close packing of isotropic particles. Considering the last state in Fig. 4a at time *t*_3_ and eliminating half of vegetation spots (cf. Fig. 4b at time *t*_1_), we use this configuration as a new initial condition for the evolution of the interaction-redistribution model Eq. (3). As the density of localised spots is decreased, the asymptotic equilibrium state changes to other types of patterns such as square, as shown in Fig. 4b. Hence, the death or removal of one or more spots affects the pattern selection process.

**Figure 5:**
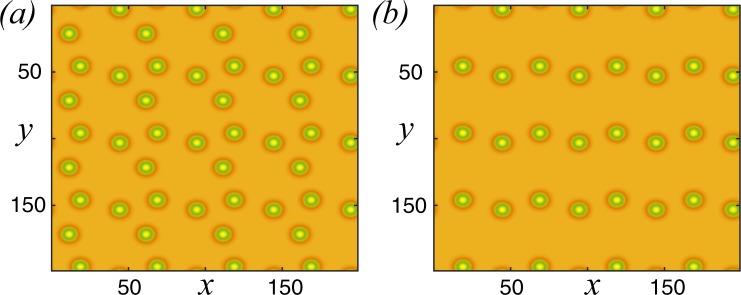
Stationary Localised patches lattice of the interaction-redistribution model Eq. (3). Parameters are *κ* = 0.6, c*D* = 0.02, Γ = 0.5, and *α* = 0.125.

  Figure 5 shows a superlattice of stable localised spots obtained by decreasing the number of spots, and by using appropriate initial conditions. This is indeed a direct consequence of the repulsive nature of the interaction. Changing the density of localised spots by removing one or more spots impacts strongly the pattern wavelength. The number of stable patterns with different symmetries and wavelengths are obviously much larger than classical Turing patterns. The obtained stationary pattern does not have an intrinsic wavelength. The characteristic wavelength depends on the localised spots density, the initial conditions, and the boundary conditions.

## Conclusion

We have explored the interaction-redistribution model for vegetation dynamics that take into account the facilitative and competitive interactions between individual plants. By carrying out numerical simulations of the integro-differential Eq. (1), we have shown that a single localised patch is a stable solution, observed far from the symmetry-breaking instability, i.e., for large values of the aridity level. However, when two or more patches are close to one another they interact in a repulsive way. This means that these vegetation spots avoid competing for their scarce resources and optimise water or nutrient uptake. We have shown that the repulsive interaction is attributed to the fact that a single patch is devoid of the damped oscillation of the tail, i.e., its tail is monotonously decreasing as a function of distance.

To understand in-depth the nature of the interaction between vegetation patches, we have focused our analysis in the vicinity of the critical point associated with bistability, where the spatiotemporal dynamics are ruled by a partial differential equation (cf. Eq. (3)). In this regime, we have derived analytically the vegetation spots interaction law and demonstrated that the interaction is always repulsive. Besides, we have shown that the repulsion interaction and boundary conditions strongly impacts the vegetation pattern selection by (i) inducing the coexistence of several patterns with different wavelengths, (ii) stabilising new extended vegetation patterns such as squares and superlattices. The proposed mechanism is general and can be applied for a broad class of out of equilibrium systems.

## Methods

Numerical simulations of models under considerations were implemented using a finite differences code with Runge-Kutta order-4 algorithm and periodic boundary conditions.

### Solvality condition

To obtain the spot interaction law, i.e., Eq. (5), we have used the Fredholm alternative method or solvability condition for linear equations^50^. Namely, considering the ansatz (4) in Eq. (3), and after linearising in *P*, one obtains the linear equation

$$\begin{array}{rcl}{\mathcal{L}}P & = & \frac{{\dot{R}}_{0}}{2}\left({\partial }_{r}{\rho }^{-}-{\partial }_{r}{\rho }^{+}\right)-2\kappa {\rho }^{+}{\rho }^{-}+3{\rho }^{+}{\rho }^{-}\bar{\rho }\\  &  & +\ \Gamma \left({\rho }^{+}{\partial }_{r}{\rho }^{-}+{\rho }^{-}{\partial }_{r}{\rho }^{+}\right)+\alpha \left({\rho }^{+}{\partial }_{r}{\rho }^{-}+{\rho }^{-}{\partial }_{r}{\rho }^{+}\right),\end{array}$$

where ${\mathcal{L}}\equiv \eta +2\kappa \bar{\rho }-3{\bar{\rho }}^{2}+D{\partial }_{rr}-\Gamma \left(\bar{\rho }{\partial }_{r}^{2}+{\partial }_{r}^{2}\bar{\rho }\right)-\alpha \left(\bar{\rho }{\partial }_{r}^{4}+{\partial }_{r}^{4}\bar{\rho }\right)$ is a linear operator, with $\bar{\rho }\equiv {\rho }^{-}+{\rho }^{+}$. To solve the former linear equation, we consider the case where localised spots are well separated from each other (*R*_0_∕*w* ≫ 1 with *w* is the localised spot waist, see Fig. 3). Thus, the distance between spots is greater than the typical size of spots. The tail of localised vegetation patch is described by the modified Bessel function of the second kind, i.e., ${\rho }^{\pm }(| r| \to \infty )\to {K}_{0}(r)={e}^{\sqrt{\eta /D}r}/\sqrt{r}$^14^. Introducing the inner product $\left\langle f| g\right\rangle ={\int }_{-\infty }^{\infty }fgdr$, the linear operator ${\mathcal{L}}$ becomes not self-adjoint (${\mathcal{L}}\ne {{\mathcal{L}}}^{\dagger }$). Numerically, we have characterised the kernel elements of the self-adjoint operator ${{\mathcal{L}}}^{\dagger }$, which are associated with the mode of interaction and translation of the centre of mass of spots^47^. Multiplying the previous equation by the interaction mode and integrating throughout the space (solvability condition), and after straightforward calculations, we obtain the spots interaction law, i.e., Eq. (5).
